# Arrhythmia and other modifiable risk factors in incident dementia and MCI among elderly individuals with low educational levels in Taiwan

**DOI:** 10.3389/fnagi.2022.992532

**Published:** 2022-12-16

**Authors:** Yen-Chang Huang, Chung-Hsiang Liu, Yu-Chi Liao, Hsin-Te Chang, Pai-Yi Chiu

**Affiliations:** ^1^Department of Psychology, College of Medical Sciences, Asia University, Taichung, Taiwan; ^2^Department of Neurology, China Medical University Hospital, College of Medicine, China Medical University, Taichung, Taiwan; ^3^Research Assistance Center, Show Chwan Memorial Hospital, Changhua, Taiwan; ^4^Department of Psychology, College of Science, Chung Yuan Christian University, Taoyuan, Taiwan; ^5^Department of Neurology, Show Chwan Memorial Hospital, Changhua, Taiwan; ^6^Department of Applied Mathematics, Tunghai University, Taichung, Taiwan

**Keywords:** arrhythmia, dementia, mild cognitive impairment, dementia, coronary heart disease, anti-lipid compounds, depression

## Abstract

**Introduction:**

There is increasing evidence that arrhythmia is a risk factor for dementia; however, it appears that arrhythmia affects the cognitive function of individuals differentially across age groups, races, and educational levels. Demographic differences including educational level have also been found to moderate the effects of modifiable risk factors for cognitive decline.

**Methods:**

This study recruited 1,361 individuals including a group of cognitively unimpaired (CU) individuals, a group of patients with mild cognitive impairment (MCI), and a group of patients with dementia with low education levels. The participants were evaluated in terms of modifiable risk factors for dementia, including arrhythmia and neuropsychiatric symptoms.

**Results:**

Cox proportional hazard regression models revealed that among older MCI patients (>75 years), those with arrhythmia faced an elevated risk of dementia. Among younger MCI patients, those taking anti-hypertensive drugs faced a relatively low risk of dementia. Among younger MCI patients, male sex and higher educational level were associated with an elevated risk of dementia. Among CU individuals, those with coronary heart disease and taking anti-lipid compounds faced an elevated risk of MCI and those with symptoms of depression faced an elevated risk of dementia.

**Discussion:**

The risk and protective factors mentioned above could potentially be used as markers in predicting the onset of dementia in clinical settings, especially for individuals with low educational levels.

## Introduction

Research has suggested that the detection of dementia in the prodromal stages (i.e., mild cognitive impairment, MCI) would enable interventions at an earlier stage (Albert et al., 2011). Recent studies have recognized the importance of detecting modifiable risk factors for dementia before the condition has progressed to a point beyond which treatment is no longer feasible. Several modifiable risk factors are reportedly associated with incident dementia, including hypertension (Hughes et al., 2020), diabetes (Dove et al., 2021), coronary heart disease (Deckers et al., 2017), dietary habits (Tsai et al., 2022), sleep disordered breathing (Leng et al., 2017), education (Roe et al., 2008; Wilson et al., 2019), and late-life depression (Brodaty and Connors, 2020). Recent studies have provided compelling evidence to support the relationship between arrhythmia and incident dementia (Liao et al., 2015; Alonso and Arenas de Larriva, 2016; Bunch, 2020). The form of arrhythmia generally attributable to incident dementia is atrial fibrillation (AF; Bunch, 2020); however, recent large-scale studies on elderly community dwellers also implicated other types of arrhythmia (Norby et al., 2021). The complex interactions among modifiable risk factors such as arrhythmia mean that the prediction of dementia based on any single factor cannot be considered reliable (Mukadam et al., 2019). Recent studies have argued that modifiable risk factors for dementia vary with the age (Miyasaka et al., 2007), race (Norby et al., 2021), and/or educational level (Mukadam et al., 2019) of the individual. For example, previous studies have shown that the correlation between arrhythmia and incident dementia tends to be stronger among elderly individuals than among those in middle age (Miyasaka et al., 2007; Rusanen et al., 2014), and the effect gradually increases with age (Miyasaka et al., 2007). One recent study has shown that the correlation between arrhythmia and incident dementia is stronger among black people than among white people (Norby et al., 2021). It has also been suggested that this correlation also varies with education level (Mukadam et al., 2019). It is crucial that researchers develop risk models that are applicable to the characteristics of the individuals (e.g., educational level and age) to whom the model is to be applied.

It is also possible that the correlation between modifiable risk factors and incident dementia varies with the disease course of dementia (Reitz et al., 2007; Yen et al., 2010; Chen et al., 2022). Note that researchers have difficulty predicting conversion from MCI to dementia, due perhaps to the heterogeneity of the modifiable risk factors (Ward et al., 2021). One recent study demonstrated that specific brain functions can moderate the effect of modifiable risk factors on cognitive performance (Chen et al., 2022). Thus, it would be reasonable to differentiate individuals according to cognitive status (e.g., cognitively unimpaired, CU, versus MCI) before evaluating the means by which they are affected by modifiable risk factors for incident dementia.

In the current study, we compared the value of arrhythmia and other modifiable risk factors for dementia in predicting the onset of incident dementia among elderly (i.e., over 75 years old) and young elderly individuals with low educational levels in Taiwan.

## Materials and methods

### Subjects

**Figure 1:**
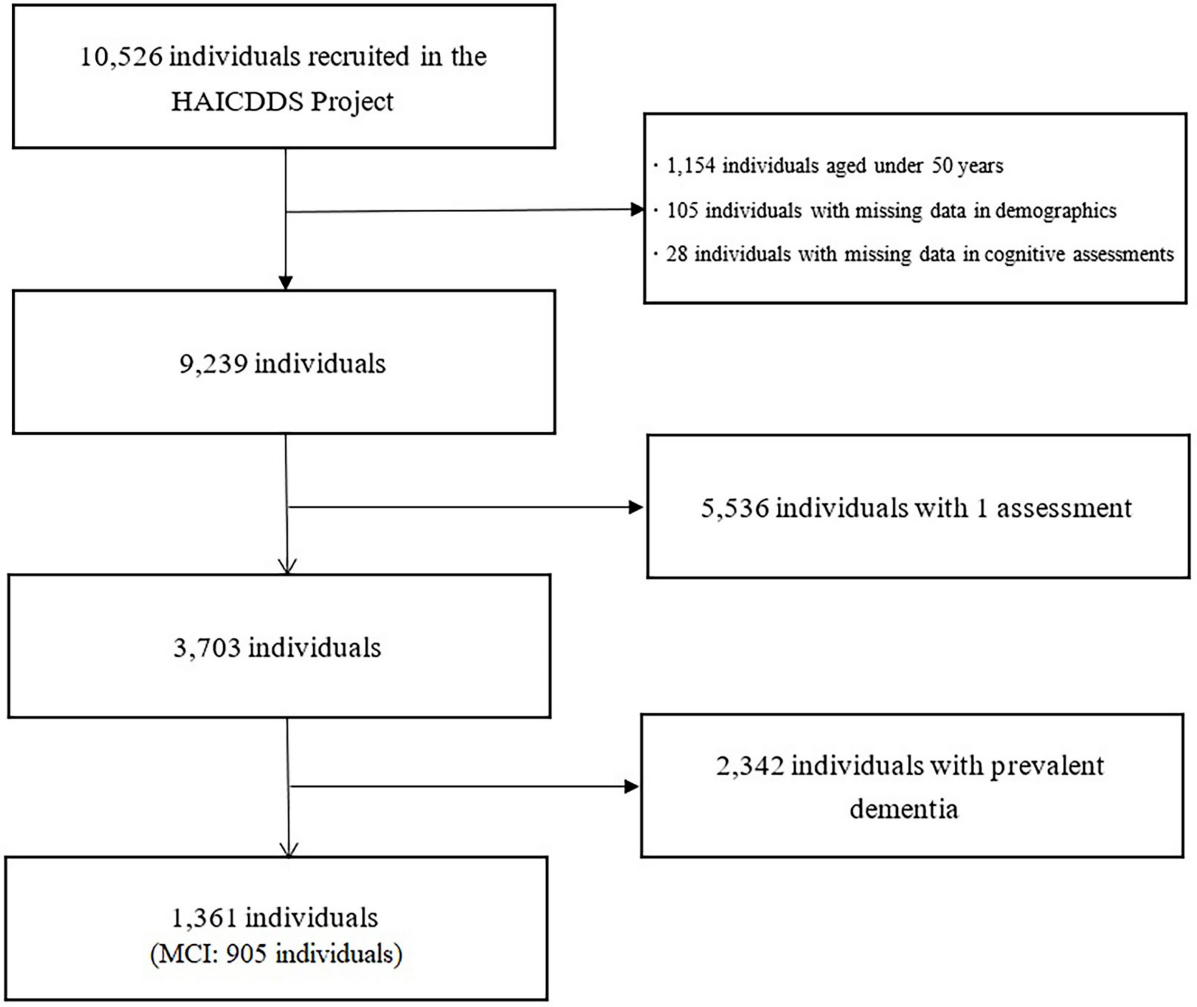
Selection process of study participants. HAICDDS, History-Based Artificial Intelligent Clinical Dementia Diagnostic System. MCI, mild cognitive impairment.

The participants were selected from a dataset built for the “History-Based Artificial Intelligent Clinical Dementia Diagnostic System (HAICDDS) Project.” As described in previous studies (Wang et al., 2013; Lin et al., 2018; Chang et al., 2020; Wang et al., 2020), the HAICDDS Project involved the retrospective analysis of a longitudinal dementia registry database from the Show Chwan Healthcare System. The aim of the project was to have participants receive regular examinations (serum tests, cognitive assessment tests, and laboratory examinations) to facilitate the early detection and prevention of dementia. At present, 10,526 participants are included in the dataset, which comprises 20,018 data points. In evaluating the predictive value of arrhythmia, we examined 1,361 individuals who underwent at least two evaluations without prevalent MCI or dementia (mean duration of follow-up: 411.45 ± 224.42 days; Figure 1). The study was approved by the Institutional Review Board of the Show Chwan Memorial Hospital. This is a retrospective study and data were processed anonymously.

### Materials and procedure

#### Assessment

Instances of incident dementia were identified through regular consensus meetings aimed at gathering the opinions of neurologists and clinical neuropsychologists. The cognitive function of the participants was evaluated by clinical neuropsychologists using the Cognitive Assessment Screening Instrument (Teng et al., 1994), Montreal Cognitive Assessment (Nasreddine et al., 2005), and Mini-Mental State Examination (Folstein et al., 1975). The daily function of participants was assessed by neurologists or clinical neuropsychologists using the Lawton’s Instrumental Activities of Daily Living (Lawton and Brody, 1969). Neuropsychiatric symptoms were assessed by clinical neuropsychologists using the Neuropsychiatric Inventory (Cummings, 1997). The criteria for a diagnosis of incident dementia followed those presented by the National Institute on Aging-Alzheimer’s Association (NIA-AA; McKhann et al., 2011). The criteria for a diagnosis of incident MCI followed those in previous studies (Albert et al., 2011). The mean age of the participants was 71.89 years, and 53.06% (*n* = 230) were female. A total of 429 participants (34.6%) displayed incident dementia at the follow-up (mean follow-up duration = 489.48 days with SD = 310.03 for participants converted from CU; mean follow-up duration = 415.57 days with SD = 218.80 for participants converted from MCI). A total of 132 individuals converted to MCI from CU (28.95%, mean follow-up duration = 415.57 days with SD = 218.80). The presence of vascular modifiable risk factors (arrhythmia, hypertension, diabetes, hypercholesterolemia, coronary heart disease, congestive heart failure) and drug interventions related to hypertension, diabetes, hypercholesterolemia, and coronary heart disease was determined in a structured interview conducted by neuropsychologists or through a review of medical charts.

#### Statistical analysis

Demographic and clinical characteristics were compared across individuals with or without incident dementia at the follow-up examination using one-way ANOVAs or chi-square tests. Survival analysis was performed using the Cox proportional hazard regression models with demographics (i.e., gender and educational levels), vascular risk factors (i.e., hypertension, diabetes, coronary artery disease, and hypercholesterolemia), drugs for controlling the vascular risk factors (i.e., taking anti-hypertensives, taking anti-diabetics, and taking anti-lipid agents), cognitive function (i.e., scores on CASI, MoCA, and MMSE), and neuropsychological symptoms (i.e., scores on the NPI) as predictors. To investigate the effects of drug therapies on vascular risk factors, we calculated the products of vascular risks factors and drugs (hypertension × taking anti-hypertensive, diabetes × taking anti-diabetic, hypercholesterolemia × taking anti-lipid agents, coronary heart disease × taking anti-hypertensive, coronary heart disease × taking anti-lipid agents) and included the products and all above-mentioned factors in the risk models. We excluded euphoria, aberrant motor behavior, and disinhibition symptoms from the analysis in the risk models due to very limited individuals displayed the symptoms (all symptoms, n = 1 in models for CU conversion to MCI or dementia; euphoria, n = 0, aberrant motor behavior and disinhibition, n = 1 in models for MCI conversion to dementia). Due to the limited number of participants with CU at baseline, we opted not to separate the risk models by age in predicting the onset of MCI or dementia among CU individuals. In the risk models, the MCI patients were divided into those over or under 75 years of age (Tables 1–3). Females were coded as 0 and males as 1. We calculated the hazard ratio (HR) for each modifiable risk factor for incident MCI or dementia. Post-hoc power analysis for the risk models was performed using G*Power 3.1 software (Faul et al., 2009).

**Table 1:** Demographic and clinical characteristics of CU participants.

	CU-CU	CU-MCI	CU-dementia	Statistical comparison
n	261	132	63	
Age	69.39 (8.12)^ab^	71.71 (7.88)^a^	74.57 (8.43)^b^	*F*_(2, 453)_ = 11.64, *p* < 0.001
Sex (% male)	52.87 (138/261)^d^	53.03 (70/132)^e^	34.92 (22/63)^de^	χ^2^_(*df* = 2, *N* = 456)_ = 7.04, *p* = 0.03
Education (years)	7.87 (4.64)^cd^	6.65 (4.63)^c^	5.10 (4.31)^d^	*F*_(2, 453)_ = 10.31, *p* < 0.001
Follow-up duration (days)	501.70 (277.27)	488.69 (253.63)	489.48 (310.03)	*F*_(2, 453)_ = 0.12, *p* = 0.89
CASI (maximum score = 100)	84.44 (9.26)^cd^	79.81 (11.70)^ce^	73.13 (16.48)^de^	*F*_(2, 453)_ = 27.98, *p* < 0.001
MoCA (maximum score = 30)	21.31 (5.24)^cd^	19.05 (6.14)^ce^	16.29 (6.25)^de^	*F*_(2, 453)_ = 22.19, *p* < 0.001
MMSE (maximum score = 30)	26.06 (3.39)^cd^	24.53 (4.23)^ce^	22.52 (5.50)^de^	*F*_(2, 453)_ = 21.99, *p* < 0.001
Hypertension	45.98 (120/261)	45.45 (60/132)	53.97 (34/63)	χ^2^_(*df* = 2, *N* = 456)_ = 1.46, *p* = 0.48
Diabetes	19.92 (52/261)	25.76 (34/132)	26.98 (17/63)	χ^2^_(*df* = 2, *N* = 456)_ = 2.52, *p* = 0.28
Hypercholesterolemia	27.97 (73/261)	30.30 (40/132)	22.22 (14/63)	χ^2^_(*df* = 2, *N* = 456)_ = 1.39, *p* = 0.50
Coronary heart disease	9.20 (24/261)	9.85 (13/132)	9.52 (6/63)	χ^2^_(*df* = 2, *N* = 456)_ = 0.05, *p* = 0.98
Arrythmia	8.81 (23/261)	10.61 (14/132)	9.52 (6/63)	χ^2^_(*df* = 2, *N* = 456)_ = 0.33, *p* = 0.85
Congestive heart failure	1.15 (3/261)	0.76 (1/132)	0 (0/63)	χ^2^_(*df* = 2, *N* = 456)_ = 0.80, *p* = 0.67
Taking anti-hypertensive	49.81 (130/261)	51.52 (68/132)	58.73 (37/63)	χ^2^_(*df* = 2, *N* = 456)_ = 1.62, *p* = 0.45
Taking anti-diabetic	16.86 (44/261)	23.48 (31/132)	26.98 (17/63)	χ^2^_(*df* = 2, *N* = 456)_ = 4.49, *p* = 0.11
Taking anti-lipid agent	32.18 (84/261)	33.33 (44/132)	30.16 (19/63)	χ^2^_(*df* = 2, *N* = 456)_ = 0.20, *p* = 0.91
Delusion	0.02 (0.37)	0.01 (0.09)	0.11 (0.63)	*F* (2, 453) = 1.83, *p* = 0.16
Hallucination	0 (0)	0.01 (0.09)	0.03 (0.25)	*F* (2, 453) = 2.36, *p* = 0.10
Agitation	0.02 (0.25)	0.01 (0.09)	0.08 (0.45)	*F* (2, 453) = 1.89, *p* = 0.15
Depression	0.5 (1.57)	0.67 (1.94)	0.83 (1.80)	*F* (2, 453) = 1.13, *p* = 0.33
Anxiety	0.23 (0.96)^a^	0.52 (1.46)^ae^	0.02 (0.13)^e^	*F* (2, 453) = 5.35, *p* = 0.005
Euphoria	0 (0)	0 (0)	0 (0)	NA
Apathy	0.05 (0.54)	0.07 (0.70)	0.06 (0.50)	*F* (2, 453) = 0.03, *p* = 0.97
Disinhibition	0 (0)	0 (0)	0 (0)	NA
Irritability	0.11 (0.64)	0.16 (0.71)	0.17 (0.64)	*F* (2, 453) = 0.32, *p* = 0.73
Aberrant behavior	0.03 (0.50)	0 (0)	0 (0)	*F* (2, 453) = 0.37, *p* = 0.69
Sleep disorder	0.86 (1.79)	1.28 (2.28)	1.10 (1.92)	*F* (2, 453) = 2.10, *p* = 0.12
Eating disorder	0.31 (1.44)	0.25 (1.18)	0.59 (1.43)	*F* (2, 453) = 1.25, *p* = 0.29

CASI: Cognitive Assessment Screening Instrument. CU: Cognitive unimpaired. CU-CU: CU individuals with stable cognitive functions. CU-dementia: CU individuals who converted to dementia. CU-MCI: CU individuals who converted to MCI. MoCA: Montreal Cognitive Assessment. MMSE: Mini-Mental State Examination. Values are expressed as mean (SD) or percentage (number/total). a: CU-CU < CU-MCI. b: CU-CU < CU-dementia. c: CU-CU > CU-MCI. d: CU-CU > CU-dementia. e: CU-MCI > CU-dementia. The maximum score is 12 for all neuropsychiatric symptoms.

**Table 2:** Demographic and clinical characteristics of patients with MCI over 75 years of age.

	MCI-non-converter	MCI-converter	Statistical comparison
n	259	249	
Age	80.18 (4.01)	81.11 (4.38)^*^	*F*_(1, 506)_ = 6.25, *p* = 0.01
Sex (% male)	46.33 (120/259)^*^	37.35 (93/249)	χ^2^_(*df* = 1, *N* = 508)_ = 4.21, *p* = 0.04
Education (years)	4.27 (4.15)	3.65 (3.95)	*F*_(1, 506)_ = 3.01, *p* = 0.08
Follow-up duration (days)	391.95 (147.55)	409.85 (216.02)	*F*_(1, 506)_ = 1.20, *p* = 0.27
CASI (maximum score = 100)	65.78 (13.87)^***^	60.20 (14.07)	*F*_(1, 506)_ = 20.26, *p* < 0.001
MoCA (maximum score = 30)	12.62 (5.48)^***^	10.43 (4.65)	*F*_(1, 506)_ = 22.95, *p* < 0.001
MMSE (maximum score = 30)	19.96 (4.59)^***^	17.91 (4.51)	*F*_(1, 506)_ = 25.75, *p* < 0.001
Hypertension	39.77 (103/259)	48.59 (121/249)^*^	χ^2^_(*df* = 1, *N* = 508)_ = 4.01, *p* = 0.045
Diabetes	20.85 (54/259)	26.51 (66/249)	χ^2^_(*df* = 1, *N* = 508)_ = 2.25, *p* = 0.13
Hypercholesterolemia	15.83 (41/259)	12.05 (30/249)	χ^2^_(*df* = 1, *N* = 508)_ = 1.51, *p* = 0.22
Coronary heart disease	6.18 (16/259)	6.83 (17/249)	χ^2^_(*df* = 1, *N* = 508)_ = 0.09, *p* = 0.77
Arrythmia	3.86 (10/259)	8.84 (22/249)^*^	χ^2^_(*df* = 1, *N* = 508)_ = 5.32, *p* = 0.02
Congestive heart failure	1.93 (5/259)	3.21 (8/249)	χ^2^_(*df* = 1, *N* = 508)_ = 0.84, *p* = 0.36
Taking anti-hypertensive	44.40 (115/259)	46.18 (115/249)	χ^2^_(*df* = 1, *N* = 508)_ = 0.16, *p* = 0.69
Taking anti-diabetic	19.69 (51/259)	23.29 (58/249)	χ^2^_(*df* = 1, *N* = 508)_ = 0.98, *p* = 0.32
Taking anti-lipid agent	21.62 (56/259)	19.68 (49/249)	χ^2^_(*df* = 1, *N* = 508)_ = 0.29, *p* = 0.59
Delusion	0.15 (0.83)	0.41 (1.47)^*^	*F*_(1, 506)_ = 6.10, *p* = 0.01
Hallucination	0.05 (0.53)	0.17 (0.81)	*F*_(1, 506)_ = 3.60, *p* = 0.06
Agitation	0.07 (0.68)	0.10 (0.71)	*F*_(1, 506)_ = 0.19, *p* = 0.66
Depression	0.56 (1.73)	0.72 (1.62)	*F*_(1, 506)_ = 1.20, *p* = 0.27
Anxiety	0.26 (1.05)	0.21 (0.91)	*F*_(1, 506)_ = 0.38, *p* = 0.54
Euphoria	0 (NA)	0 (NA)	NA
Apathy	0.17 (0.84)	0.46 (1.39)^**^	*F*_(1, 506)_ = 8.34, *p* = 0.004
Disinhibition	0 (NA)	0 (NA)	NA
Irritability	0.49 (1.49)	0.32 (1.08)	*F*_(1, 506)_ = 2.14, *p* = 0.14
Aberrant behavior	0.03 (0.56)	0 (0)	*F*_(1, 506)_ = 0.96, *p* = 0.32
Sleep disorder	0.72 (1.55)	1.06 (1.95)^*^	*F*_(1, 506)_ = 4.93, *p* = 0.03
Eating disorder	0.44 (1.51)	0.46 (1.50)	*F*_(1, 506)_ = 0.03, *p* = 0.87

CASI: Cognitive Assessment Screening Instrument. MoCA: Montreal Cognitive Assessment. MMSE: Mini-Mental State Examination. Values are expressed as mean (SD) or percentage (number/total). ^*^ *p* < 0.05. ^**^ *p* < 0.01. ^***^ *p* < 0.001. The maximum score is 12 for all neuropsychiatric symptoms.

**Table 3:** Demographic and clinical characteristics of patients with MCI under 75 years of age.

	MCI-non-converter	MCI-converter	Statistical comparison
n	280	117	
Age	66.30 (6.01)	67.85 (5.41)^*^	*F*_(1, 395)_ = 5.82, *p* = 0.02
Sex (% male)	40.71 (114/280)	56.41 (66/117)^**^	χ^2^_(*df* = 1, *N* = 397)_ = 8.20, *p* = 0.004
Education (years)	6.98 (4.23)	6.52 (4.21)	*F*_(1, 395)_ = 0.95, *p* = 0.33
Follow-up duration (days)	440.02 (267.03)	427.75 (225.06)	*F*_(1, 395)_ = 0.19, *p* = 0.66
CASI (maximum score = 100)	77.47 (11.82)^***^	68.72 (16.35)	*F*_(1, 395)_ = 35.67, *p* < 0.001
MoCA (maximum score = 30)	18.29 (6.18)^***^	14.36 (6.13)	*F*_(1, 395)_ = 31.53, *p* < 0.001
MMSE (maximum score = 30)	23.50 (4.10)^***^	20.85 (5.31)	*F*_(1, 395)_ = 28.76, *p* < 0.001
Hypertension	48.21 (135/280)	47.86 (56/117)	χ^2^_(*df* = 1, *N* = 397)_ = 0.004, *p* = 0.95
Diabetes	27.14 (76/280)	35.04 (41/117)	χ^2^_(*df* = 1, *N* = 397)_ = 2.48, *p* = 0.12
Hypercholesterolemia	31.43 (88/280)	25.64 (30/117)	χ^2^_(*df* = 1, *N* = 397)`_ = 1.32, *p* = 0.25
Coronary heart disease	7.86 (22/280)	7.69 (9/117)	χ^2^_(*df* = 1, *N* = 397)_ = 0.003, *p* = 0.96
Arrythmia	11.07 (31/280)	11.97 (14/117)	χ^2^_(*df* = 1, *N* = 397)_ = 0.07, *p* = 0.80
Congestive heart failure	3.93 (11/280)	4.27 (5/117)	χ^2^_(*df* = 1, *N* = 397)_ = 0.03, *p* = 0.87
Taking anti-hypertensive	53.21 (149/280)	51.28 (60/117)	χ^2^_(*df* = 1, *N* = 397)_ = 0.12, *p* = 0.73
Taking anti-diabetic	24.29 (68/280)	31.62 (37/117)	χ^2^_(*df* = 1, *N* = 397)_ = 2.28, *p* = 0.13
Taking anti-lipid agent	35.71 (100/280)	42.74 (50/117)	χ^2^_(*df* = 1, *N* = 397)_ = 1.73, *p* = 0.19
Delusion	0.19 (1.04)	0.18 (1.18)	*F*_(1, 395)_ = 0.01, *p* = 0.91
Hallucination	0.14 (0.85)	0.15 (1.18)	*F*_(1, 395)_ = 0.03, *p* = 0.86
Agitation	0.16 (0.96)	0.18 (0.88)	*F*_(1, 395)_ = 0.03, *p* = 0.86
Depression	1.14 (2.29)	1.29 (2.41)	*F*_(1, 395)_ = 0.35, *p* = 0.56
Anxiety	0.52 (1.73)	0.57 (1.87)	*F*_(1, 395)_ = 0.08, *p* = 0.78
Euphoria	0.03 (0.36)	0 (0)	*F*_(1, 395)_ = 0.55, *p* = 0.46
Apathy	0.44 (1.65)	0.53 (1.86)	*F*_(1, 395)_ = 0.21, *p* = 0.64
Disinhibition	0.07 (0.62)	0.06 (0.56)	*F*_(1, 395)_ = 0.03, *p* = 0.86
Irritability	0.64 (1.69)	0.45 (1.45)	*F*_(1, 395)_ = 1.04, *p* = 0.31
Aberrant behavior	0.08 (0.76)	0.05 (0.56)	*F*_(1, 395)_ = 0.12 *p* = 0.73
Sleep disorder	1.21 (2.26)	1.06 (1.99)	*F*_(1, 395)_ = 0.38, *p* = 0.54
Eating disorder	0.54 (1.91)	0.60 (1.76)	*F*_(1, 395)_ = 0.08, *p* = 0.77

CASI: Cognitive Assessment Screening Instrument. MoCA: Montreal Cognitive Assessment. MMSE: Mini-Mental State Examination. Values are expressed as mean (SD) or percentage (number/total). ^*^ *p* < 0.05. ^**^ *p* < 0.01. ^***^ *p* < 0.001. The maximum score is 12 for all neuropsychiatric symptoms.

## Results

Post-hoc power analyses revealed that the power of all risk models was sufficient (all exceeding 0.99).

### From CU to MCI or dementia

**Figure 2:**
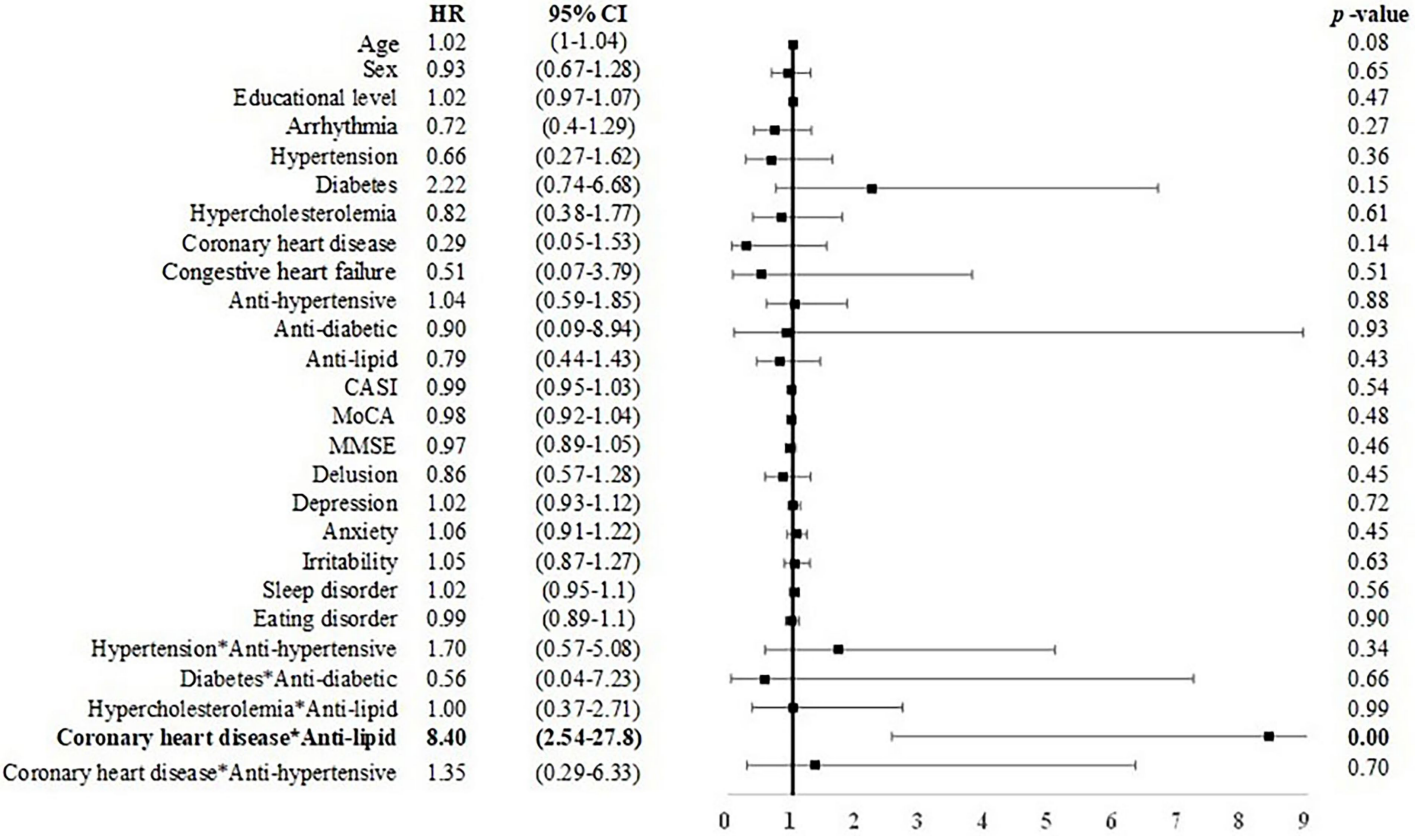
Risk of incident MCI among CU individuals. CASI, Cognitive Assessment Screening Instrument; MMSE, Mini-Mental State Examination, CI, confidence interval; HR, hazard ratio. Male sex was coded as 1 and female as 0 in the model. Bold font indicates statistically significant.

**Figure 3:**
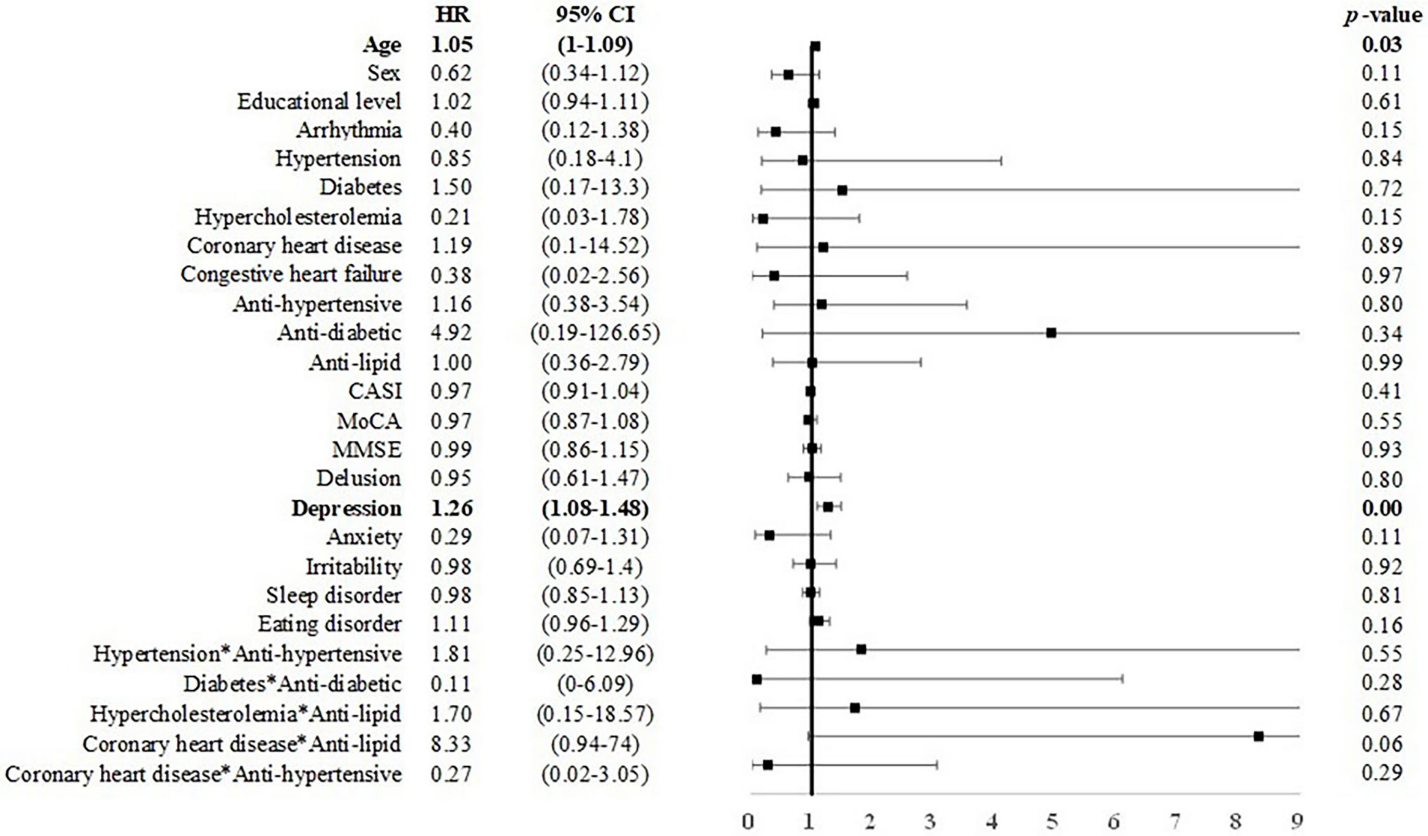
Risk of incident dementia among CU individuals. Note and abbreviations as those used in Figure 2.

Among elderly CU individuals, those with coronary heart disease and taking anti-lipid agents was associated with an elevated risk of MCI (*B* = 2.128, HR = 8.40, CI: 2.54–27.80). Age (*B* = 0.05, *p* < 0.05, HR = 1.05, CI = 1.00–1.09) and depression symptoms (*B* = 0.24, *p* < 0.01, HR = 1.27, CI = 1.08–1.48) were positively associated with incident dementia (Figures 2, 3). No other factors were predictive of incident MCI or incident dementia (*p* = 0.21–0.99). Dividing CU individuals into those under or over 75 years of age in post-hoc analysis revealed similar but statistically insignificant trends for the predictors, due perhaps to the limited sample size.

### From MCI to dementia

**Figure 4:**
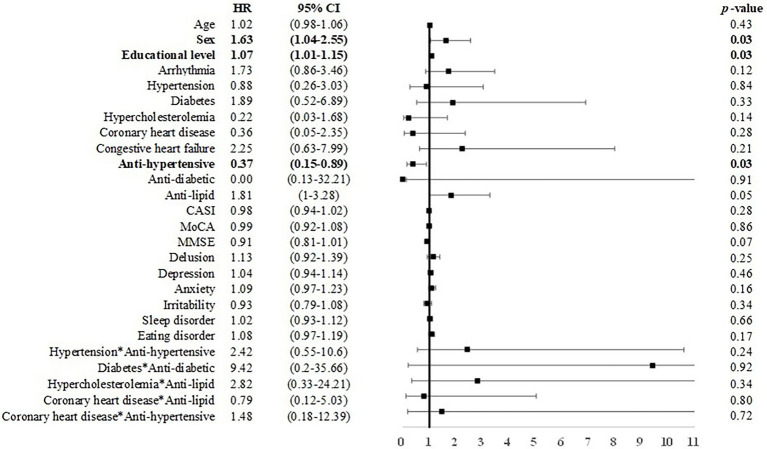
Risk of incident dementia among individuals with MCI under 75 years of age. Note and abbreviations as those used in Figure 2.

**Figure 5:**
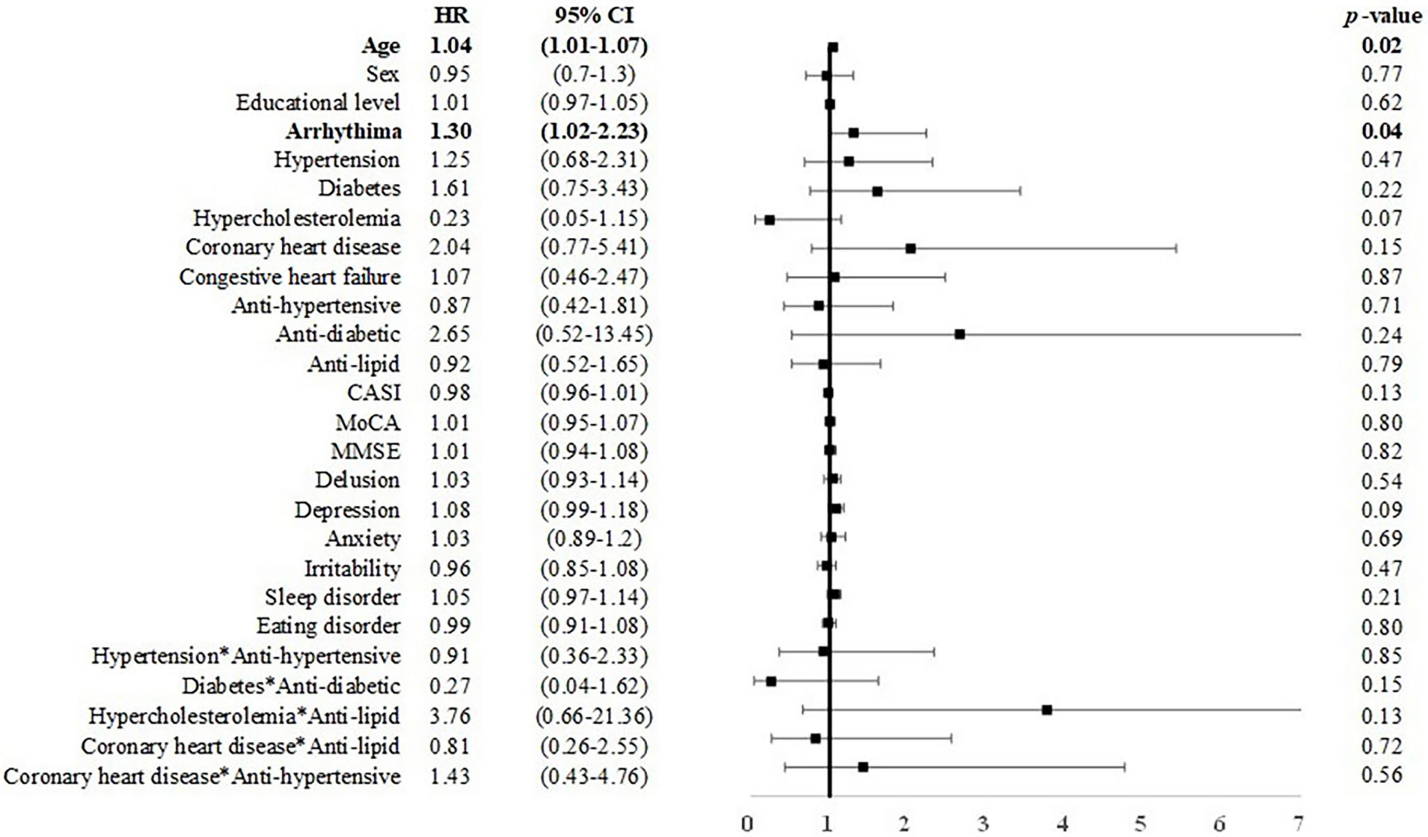
Risk of incident dementia among individuals with MCI over 75 years of age. Note and abbreviations as those used in Figure 2.

Among individuals with MCI under 75 years of age, male sex was associated with incident dementia (*B* = 0.46, *p* < 0.05, HR = 1.58, CI = 1.01–2.48). Anti-hypertensive use was associated with a reduced risk for dementia (*B* = −1.00, HR = 0.37, CI: 0.15–0.89; Figure 4). Among individuals with MCI over 75 years of age, age (*B* = 0.04, HR = 1.03, CI: 1.00–1.07) and arrhythmia (*B* = 0.14, HR = 1.30, CI: 1.02–2.23) were associated with an elevated risk for dementia (Figure 5).

### Discussion

This retrospective study investigated the predictive value of arrhythmia and other risk factors for MCI and dementia among elderly individuals with a low educational level. Our findings revealed that arrhythmia was a risk factor for dementia only among MCI patients in the older group (>75 years). Among CU individuals, depression was a risk factor for incident dementia. Among MCI individuals under 75 years of age, anti-hypertensives use was associated with a reduced risk of dementia, whereas male sex and higher educational level were correlated with an elevated risk of dementia.

Recent studies have reported that demographic differences, such as education level, can moderate the effect of modifiable risk factors for incident dementia (Ericsson et al., 1991; Mukadam et al., 2019). The effect of arrhythmia on incident dementia could perhaps be associated with the low education level of individuals in our sample. Other factors may be more important than arrhythmia in predicting the onset of dementia among younger MCI patients.

Previous studies have proposed that arrhythmia (particularly AF type) can lead to the formation of emboli in the heart (Liao et al., 2015; Alonso and Arenas de Larriva, 2016; Bunch, 2020; Norby et al., 2021), which can travel to the brain to cause stroke. Asymmetric ventricular dyssynchrony can lead to an increase in myocardial wall thickness and alter blood flow in the myocardium, with corresponding effects on cerebral hypoperfusion and cognitive decline (Norby et al., 2021). This has led to the adoption of arrhythmia as a risk factor for dementia (particularly VaD). One previous study reported that the correlation between arrhythmia and stroke increases with age (Björck et al., 2013). Previous studies have consistently reported a strong correlation between hypertension and stroke (Wolf et al., 1991; Odonnell et al., 2010; Freedman et al., 2016). Nonetheless, the predictive value of hypertension on dementia becomes similar to that of arrhythmia as the individual ages. The correlation between arrhythmia and dementia also increases with age (Rusanen et al., 2014). These findings indicate that among MCI patients, arrhythmia is an important risk factor for dementia in elderly individuals and that the importance increases with the age of the individual.

MCI is a prodromal state of dementia associated with various forms of brain dysfunction, including changes in white matter and atrophy in the brain (Kim et al., 2021). Nonetheless, the daily functioning of MCI patients is largely unaffected. One recent study reported that brain functioning can modulate the relationship between modifiable risk factors for dementia and cognitive function (Chen et al., 2022). Older MCI patients with compromised brain function may be more vulnerable than their younger counterparts to emboli or brain hypoperfusion resulting from arrhythmia.

Researchers have suggested that treatments for coronary heart disease reduce the risk of dementia (Justin et al., 2013; Nguyen et al., 2022). Previous studies have reported inconsistent results pertaining to a correlation between statins and the risk of dementia (Zhang et al., 2018; Lu and Li, 2021; Zhou et al., 2021). Most research has indicated that the benefits of taking statins outweigh the risks (Poly et al., 2020; Zhou et al., 2021); however, researchers have pointed out a non-linear relationship between statin dosage and the risk of developing dementia (Zhang et al., 2018). The inconsistent findings may also be due to ethnic differences and/or comorbidities profiles of the samples. In the current study, we also found that CU individuals with coronary heart disease who had been taking anti-lipid compounds (95.5% were statins in this study) faced an elevated risk for dementia. Note however that we did not collect information related to statin dosage. Future studies should investigate this issue.

Hypertension has been identified as a risk factor for VaD (Joas et al., 2012). Our findings are in line with previous work indicating that the management of hypertension is crucial to the prevention of VaD (Guo et al., 2021). We also determined that younger male MCI patients faced an elevated likelihood of developing dementia, which may be due to the correlation between male sex and VaD, particularly among younger elderly individuals (Liu et al., 1998). In contrast, taking anti-hypertensives was a protective factor among younger MCI patients in this study. These results are in accordance with previous studies indicating that managing blood pressure can reduce the risk of dementia, particularly among younger elderly individuals (Joas et al., 2012).

Among younger MCI patients in this study, higher educational level was associated an elevated risk of dementia. The result is inconsistent with most previous findings (Roe et al., 2008; Stern et al., 2020). Note however that among the elderly in Taiwan, there is a notable gender difference in educational level (Ministry of the Interior, 2021); i.e., the education level is generally higher among elderly males. Thus, the elevated risk faced by individuals with higher educational level might be explained by this gender difference.

It has been argued that subjective memory complaints (SMC) are an early sign of Alzheimer’s disease (Jessen et al., 2022), whereas other studies have proposed that SMC among elderly individuals is associated with emotional symptoms (e.g., Buckley et al., 2013). In the current study, we determined that incident dementia is associated with the symptoms of depression. These findings provide further evidence that emotional symptoms are predictive of cognitive changes. According to the “vascular depression” hypothesis of late-life depression, the depressive symptoms exhibited by elderly individuals are mediated through cerebrovascular changes (Alexopoulos et al., 1997; Taylor et al., 2003). Our results may reflect the interaction between vascular etiologies and AD-related neuropathologies commonly observed among the elderly (Ho et al., 2022). Previous studies have reported a higher prevalence of psychiatric symptoms among individuals in low-or middle-income countries (Guerra et al., 2009; Mukadam et al., 2019). The predictive value of emotional symptoms for incident dementia among CU individuals may have clinical importance in the treatment of symptoms and the prevention of cognitive decline (Brodaty and Connors, 2020).

This study shed light on the mechanisms underlying cognitive decline among elderly CU and MCI individuals. Note however that these findings were subject to various limitations. First, the participants were recruited from a clinical setting, such that the findings are not necessarily applicable to the community at large. Second, the sample size in the current study was relatively limited. Future research using a larger heterogeneous sample will be required to further unravel the effects of the modifiable factors and potentially complex interactions among these factors. Third, vascular risks were evaluated using self-or informant-reported data or a review of medical charts. As a result, we were unable to investigate the severity of vascular factors or dosages and adherence to treatment. Furthermore, we did not collect data related to treatments for arrhythmia and congestive heart failure. Fourth, we did not address all of the factors associated with incident dementia in later life. For example, we disregarded the fact that *APOE* genotype has previously been associated with incident dementia (Livingston et al., 2020; Pillai et al., 2021). Further research will be required to investigate the degree to which awareness of vascular risks and factors (e.g., physical activities, social isolation, and air pollution exposure) affects the predictions of dementia. Fifth, the mean age of the participants in this study was older than those in many other studies, and their educational levels were lower. Thus, these results should be applied with caution to younger or more highly educated populations. Sixth, we evaluated only the effects of modifiable risk factors for all-cause dementia, and participants were recruited only in Taiwan. Cross-validation in different countries will be required to confirm our findings.

In conclusion, this study identified arrhythmia as a predictive factor for incident dementia among MCI patients aged over 75 years. Anti-hypertensive use was associated with a reduced risk of dementia among MCI patients aged under 75 years. Practitioners treating CU individuals should exercise caution in assessing cognitive decline while treating coronary artery disease with anti-lipid agents. It appears that depressive symptoms may be important in predicting dementia among CU individuals. These findings could be used as markers in predicting dementia in clinical settings, particularly among elderly individuals with a low educational level.

## Data availability statement

The data analyzed in this study is subject to the following licenses/restrictions: Data available on request due to privacy/ethical restrictions. Requests to access these datasets should be directed to H-TC, changht@cycu.edu.tw.

## Ethics statement

The studies involving human participants were reviewed and approved by Institutional Review Board of the Show Chwan Memorial Hospital. Written informed consent for participation was not required for this study in accordance with the national legislation and the institutional requirements.

## Author contributions

Y-CH, C-HL, P-YC, and H-TC: conception and design of study and acquisition of data. Y-CH, Y-CL, P-YC, and H-TC: analysis and interpretation of data. Y-CH and H-TC: drafting the manuscript. H-TC and P-YC: revising the manuscript critically for important intellectual content. Y-CH, C-HL, Y-CL, P-YC, and H-TC: approval of the version of the manuscript. All authors contributed to the article and approved the submitted version.

## Conflict of interest

The authors declare that the research was conducted in the absence of any commercial or financial relationships that could be construed as a potential conflict of interest.

## Publisher’s note

All claims expressed in this article are solely those of the authors and do not necessarily represent those of their affiliated organizations, or those of the publisher, the editors and the reviewers. Any product that may be evaluated in this article, or claim that may be made by its manufacturer, is not guaranteed or endorsed by the publisher.
